# Electron Bifurcation Arises from Emergent Features of Multicofactor Enzymes

**DOI:** 10.1021/acsomega.5c13233

**Published:** 2026-05-13

**Authors:** Anna Wójcik-Augustyn, Łukasz Bujnowicz, Artur Osyczka, Marcin Sarewicz

**Affiliations:** Department of Molecular Biophysics, Faculty of Biochemistry, Biophysics and Biotechnology, 98817 Jagiellonian University in Kraków, Gronostajowa 7, Kraków 30-387, Poland

## Abstract

Quinone-based electron bifurcation (QBEB) catalyzed by cytochrome *bc*_1_ (cyt. *bc*_1_) plays a critical role in maximizing the efficiency of biological energy conversion. The canonical QBEB model, grounded in equilibrium redox potentials, dictates the order of QBEB steps with initial endergonic reduction of a high-potential iron–sulfur cluster (2Fe2S) by quinol followed by exergonic reduction of low-potential heme *b*_L_ by semiquinone (SQ). However, this concept falls short in explaining several experimental observations, including intermediate semiquinone spin-coupled to 2Fe2S (SQ-2Fe2S^red^) and the absence of short-circuiting. The current DFT calculations, performed on large cluster models of cyt. *bc*_1_, encompassing both 2Fe2S and heme *b*_L_, identified the location of donor (HOMO) and acceptor (LUMO) orbitals along with the previously unconsidered microstates to reveal that QBEB is an emergent property of an integrated system of redox cofactors where transient charge separations dynamically modulate electron affinities. In this system, electron transfer initiates preferentially toward heme *b*_L_, indicating a departure from the conventional sequence proposed by the canonical QBEB. Based on this finding, we introduce an emergent electron transfer (EMET) model of QBEB and demonstrate that its assumptions are supported by electron paramagnetic resonance spectroscopy data. Unlike the canonical QBEB, EMET proposes a relatively flat energy profile for the QBEB that accommodates stable SQ-2Fe2S^red^ and explains suppression of short circuits without additional assumptions. It highlights the importance of state-dependent electrostatic interactions in shaping electron transfer pathways. In general, the concept of emergence inherent to EMET offers a mechanistic framework applicable to a broad range of multicofactor redox enzymes beyond cyt. *bc*_1_.

## Graphical abstract

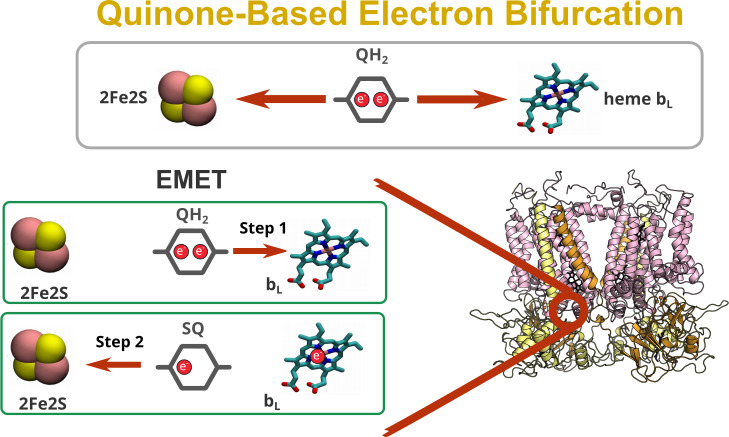

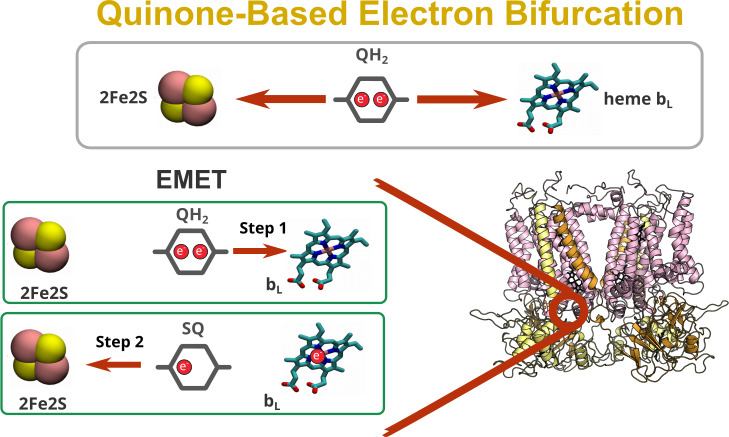

## Introduction

The quinone-based electron bifurcation (QBEB) is a crucial process that maximizes the efficiency of energy conversion during respiration and photosynthesis.^1–5^ Although flavin-based electron bifurcation (FBEB) has also been identified in some enzymes,^6,7^ the present work focuses on the mechanism of electron bifurcation catalyzed by cytochromes (cyt.) *bc* of the Rieske/*b* family, including cytochrome *bc*_1_ (cyt. *bc*_1_) in bacteria, mitochondrial complex III, and cyt. *b*_6_ *f* in plants.^2,8,9^ These enzymes share a similar homodimeric structure across all organisms, exhibiting nearly identical core compositions (Figure 1A) and spatial arrangements of redox cofactors (Figure 1B) as well as two catalytic sites, denoted Q_o_ and Q_i_, located on opposite sides of the membrane (o for outer and i for inner). In these sites, oxidation of quinol (QH_2_) and reduction of quinone (Q) take place, respectively.^2,10,11^ In each monomer, the cofactors are organized into two electron-transfer branches: the low-potential chain, consisting of two *b* hemes with relatively low redox potentials (usually ≪ + 100 mV), and the high-potential chain, consisting of the 2Fe2S cluster and heme *c*_1_, both having relatively high positive redox potentials (>+200 mV). The catalytic quinol oxidation site Q_o_ is located between these low- and high-potential chains (Figure 1C). Upon binding to this site, QH_2_ becomes flanked by two redox cofactors: the Rieske iron–sulfur cluster (2Fe2S) from the high-potential chain and heme *b*_L_ from the low-potential chain. A characteristic feature of the Rieske cluster is its unique coordination of the 2Fe2S cluster by two cysteine residues and two histidine residues, which couples its redox transition to protonation/deprotonation of one of the histidines. This contrasts with plant-type [2Fe-2S] clusters, which are coordinated exclusively by four cysteines.^12^

**Figure 1:**
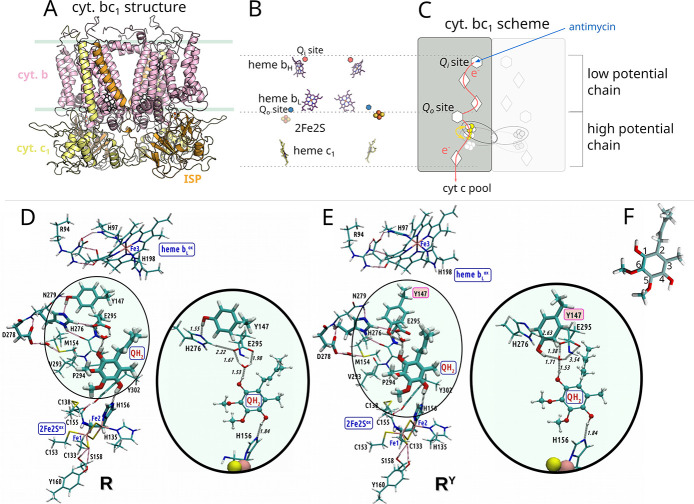
Overview of the structural elements of cyt. *bc*_1_. (A) The crystallographic model of the cyt. *bc*_1_ dimer from *Rhodobacter capsulatus* (PDB ID: 1ZRT). Each monomer consists of 3 catalytic subunits: cytochrome *b* (cyt. *b*), cytochrome (cyt. *c*_1_), and iron–sulfur protein (ISP). (B) The spatial arrangement of redox-active cofactors: hemes *b*_L_, *b*_H_ of cyt. *b*, 2Fe2S of ISP, and heme *c*_1_ of cyt. *c*_1_. Location of Q_i_ and Q_o_ sites is depicted as red and blue hexagons, respectively. (C) General pathways for electrons (*red arrows*) released upon oxidation of QH_2_ at Q_o_ (*white hexagon*). QBEB directs one electron to the high potential chain (consisting of 2Fe2S, *c*_1_ and *c*) and one electron to the low potential chain (consisting of hemes *b*_L_, *b*_H_ and Q_i_). *White diamonds* denote hemes, and *circles* denote 2Fe2S. Yellow arrow indicates movement of the head domain of ISP (ISP-HD) containing 2Fe2S between cyt. *b* and cyt. *c*_1_. The blue arrow shows where the inhibitor antimycin binds. For simplicity, the scheme shows ET in one monomer. (D, E) Optimized initial structures of the QM model with two possible conformations of cyt. *b*:Y147: (D) **R** with Y147 directed toward cyt. *b*:H276, and (E) **R** ^**Y**^ with Y147 directed toward bound QH_2_. Fragments of the models, highlighting the different conformations of the Y147 side chain, are magnified. (F) The quinone moiety with the carbon atom numbering of the ring indicated.

In cyt. *bc*_1_, QBEB relies on the two-electron oxidation of QH_2_, with each electron diverging into a different direction by transfer to distinct electron acceptors: oxidized heme *b*_L_ (*b*_L_^ox^) and oxidized 2Fe2S (2Fe2S^ox^):

$$\mathrm{heme}\;b_{L}^{\mathrm{ox}}\leftarrow {\mathrm{QH}}_{2}\to 2\mathrm{Fe}2S^{\mathrm{ox}}$$

A one-to-one correlation between the number of electrons transferred from QH_2_ to heme *b*_L_^ox^ and to 2Fe2S^ox^ is expected to ensure maximal energy conservation efficiency while preventing direct or quinone-mediated electron exchange between heme *b*_L_ and 2Fe2S. Although this process appears conceptually simple, it has never been reproduced in synthetic systems.^13^ The QBEB reaction allows the conversion of stoichiometry from two-electron (ubiquinol) to one-electron carriers.

The general framework for QBEB mechanism, originally proposed by Mitchell, was based on the formalism of equilibrium electrochemistry, i.e., on the equilibrium redox midpoint potentials.^14,15^ It describes QBEB as two consecutive one-electron steps, with the Q/QH_2_ redox reaction divided into two half-reactions involving the SQ/QH_2_ and Q/SQ redox couples (where SQ and Q denote semiquinone and quinone, respectively). These couples are proposed to differ significantly in their redox midpoint potentials (*E*_m_), with the SQ/QH_2_ couple being very high-potential (>400 mV) and the Q/SQ couple being very low-potential (∼−200 mV).^16,17^ This suggests that QBEB is driven by large (more than 600 mV) differences between these couples.^17^ Accordingly, QBEB is assumed to begin with an uphill oxidation of QH_2_ to SQ by 2Fe2S (*E*_m_^2Fe2S^ ∼ 300 mV) followed by downhill oxidation of SQ to Q by heme *b*_L_ (*E*_m_^*b*L^ ∼ −120 mV) and the first uphill step is generally considered as a rate-limiting step in the cyt. *bc*_1_ catalysis. This specific sequence of QH_2_ oxidation, first by 2Fe2S^ox^ and then by heme *b*_L_^ox^, derived from consideration of *E*_m_ values for the immediate acceptors (i.e., 2Fe2S and heme *b*_L_) and the Q/SQ/QH_2_ triad, has become the canonical QBEB mechanism and the basis for interpreting both experimental and computational studies. Hereafter, this model is termed the canonical QBEB mechanism.

Several important consequences follow from the canonical QBEB. First, SQ is expected to be very unstable and transient, as the first step (oxidation of QH_2_ to SQ by 2Fe2S) is endergonic, while the second (oxidation of SQ to Q by heme *b*_L_) is exergonic.^17^ Consequently, SQ is presumed to be highly unstable and potentially a substrate for superoxide radical formation due to low *E*_m_ of Q/SQ couple.^18^ This inherent instability may explain the long-standing challenges associated with the direct detection of SQ at the Q_o_ site. Second, considering the *E*_m_ values of heme *b*_L_, 2Fe2S, and the Q/SQ/QH_2_ triad, the overall reaction should, under certain conditions, be susceptible to internal, quinone-mediated electron exchange between the chains, that is, quinone-mediated electron transfer from heme *b*_L_^red^ to 2Fe2S.^2,4^ This issue becomes especially problematic in understanding the inhibition of cyt. *bc*_1_ by antimycin.^19,20^

Antimycin binds specifically and tightly to quinone reduction site Q_i_, thereby preventing quinone binding at this site. As a result, the electron transfer from heme *b*_H_ to quinone is blocked. This leads to severe inhibition of catalytic turnover, as the rate of cyt. *c* reduction decreases dramatically, rendering the enzyme virtually inactive under steady-state conditions.

According to the canonical QBEB, once antimycin blocks the Q_i_ site, two consecutive cycles of QH_2_ oxidation at the Q_o_ site should lead to the reduction of both hemes *b*_H_ and *b*_L_. Subsequent oxidation of QH_2_ by 2Fe2S^ox^ would then be followed by the thermodynamically favorable reduction of SQ back to QH_2_ by electron transfer (ET) from heme *b*_L_^red^. As a consequence, QBEB would be bypassed with both electrons from QH_2_ flowing exclusively through the high-potential chain. This possible reaction is referred to as short circuit reaction.^4,17^ However, so far, such events have remained experimentally undetectable. On the other hand, under nonequilibrium conditions in the presence of antimycin, relatively large amounts of SQ spin-coupled to 2Fe2S^red^ (SQ–2Fe2S^red^) and small amounts of SQ free radical signal have been detected.^21–25^ Yet, the roles of these species in QBEB remain poorly understood. While the presence of the SQ signal can still be explained within the framework of the canonical QBEB, the problem arises when considering the substantial population of SQ spin-coupled to reduced 2Fe2S (SQ–2Fe2S^red^). These unresolved observations and the lack of expected short circuits have long hindered a full mechanistic understanding of QBEB in cyt. *bc*_1_.

The issue associated with the inhibitory effect of antimycin is particularly important, considering the fact that this inhibitor has been widely used for years in experiments performed to understand the mechanism of QBEB. Simple models based solely on *E*_m_s face problems in explaining the inhibition of QH_2_ oxidation and the lack of internal short circuits. Over the years, various models have been proposed to overcome these problems, yet the aforementioned issues have never been satisfactorily addressed.^2^ Consequently, several, often mutually exclusive, so-called gating mechanisms of QBEB coexist,^17,26–32^ raising the concern that a fundamental principle underlying efficient QH_2_ oxidation by cyt. *bc*_1_ might still be missing and that a complete description of Q_o_ catalysis has yet to be formulated.^5^

Here, we employed quantum mechanical (QM) calculations to analyze the QBEB in cyt. *bc*_1_ at the level of the electronic structure, allowing us to follow the progress of the key steps of the reaction. We also aimed to explain the mechanism of formation and the role of SQ–2Fe2S^red^ in suppressing short circuits and the mechanism of antimycin inhibition of QH_2_ oxidation at the Q_o_ site of cyt. *bc*_1_ from purple bacteria *Rhodobacter capsulatus*. To this end, we performed density functional theory (DFT) analyses on models containing both heme *b*_L_ and 2Fe2S cofactors as inseparable components of the QBEB reaction. To the best of our knowledge, this approach has not been previously applied in studies of the QBEB mechanism in cyt. *bc*_1_ and was chosen to avoid or at least minimize the artificial selection of the reaction sequence inherent in models that isolate individual one-electron reactions. Our *ab initio* approach leads us to the demonstration that all redox-active elements act as an integrated system of cofactors, whose behavior cannot be reproduced by two separate models comprising the quinone molecule and either heme *b*_L_ or 2Fe2S. In particular, we show that the initiation of QBEB deviates significantly from the canonical QBEB and proceeds through previously unconsidered microstates. Based on the calculations, we developed a model of QBEB and analyzed its predictions with existing data and the results of newly performed experiments. We believe that these results help to reconcile the apparent discrepancies between reports detecting semiquinone intermediates within the Q_o_ site of cyt. *bc*_1_ and cyt. *b*_6_ *f* while also explaining the absence of short circuiting and the mechanism of antimycin inhibition. If our model correctly describes the QBEB mechanism, it also underscores the potential limitations of using equilibrium-determined *E*_m_ values to construct each step of the reaction. Rather, charge separations, resulting from ET and PT within the protein interior, can modify interactions (Coulombic, for example) between cofactors, leading to state-dependent changes in their electron affinities.

Although our proposition represents a new hypothesis that requires extensive future validation, it offers a unified explanation for several long-standing challenges. We propose that this model provides mechanistic insight into the efficiency of the QBEB and may guide the design of synthetic systems.

## Materials and Methods

### QM Model of the Q_o_ Active Site

The cluster model of the Q_o_ active site was constructed based on the crystallographic structure of cyt. *bc*_1_ from *R. capsulatus* (PDB code: 1ZRT).^33^ Stigmatellin bound at the Q_o_ site was replaced with ubiquinol, preserving the position of the hydrophobic tail, and two water molecules were added based on molecular dynamics simulations.^34^ The structure containing stigmatellin was selected as the model for constructing the active state **R**, following suggestions that stigmatellin at Q_o_ mimics the enzyme–substrate complex between quinol and 2Fe2S,^35^ consisting of 399 atoms and including the following elements: heme *b*_L_, ubiquinol (with its hydrophobic tail truncated to one isoprene unit), two water molecules, the 2Fe2S cluster, eight residues from chain E (C133, H135, C138, C153, C155, H156, S158, Y160), and 12 residues from chain P (R94, H97, Y147, M154, H198, H276, D278, N279, V293, P294, E295, Y302). The residues from chain E (C133, H135, C138, C153, S158, Y160) and chain P (R94, H97, Y147, M154, H198, H276, Y302) were included with their amino and carbonyl moieties of the protein backbone replaced by hydrogen atoms. The protein fragments C155–H156 (chain E) and D278–N279, V293–P294–E295 (chain P) were included along with the peptide bonds connecting them, with the corresponding CO and NH groups of the protein backbone substituted by hydrogen atoms (i.e., for C155, D278, and V293, the NH groups were replaced, and for H156, N279, and E295, the CO groups were replaced).

Protonation states of the residues were assigned using program Propka 3.1,^36,37^ validated by PypKa 2.10.0.^38^ For the H156 residue, which coordinates the 2Fe2S cluster and is hypothesized to act as a direct proton acceptor during QH_2_ oxidation, two protonation states were tested. In the first state, the H156 ligand was deprotonated and ready to accept a proton, while in the second state, H156 was protonated at the N_τ_ atom. The protonation state of the second histidine ligand H135 does not depend on the redox state of the cluster and remains protonated.^39,40^ To maintain the rigidity of the protein backbone, geometry optimizations of all structures considered in the present study were performed with constraints imposed on the hydrogen atoms replacing protein backbone moieties and on the carbon atoms that are part of the protein backbone.

### QM Model of the Active Q_i_ Active Site

The QM models of the Q_i_ site were constructed based on the crystallographic structure of cyt. *bc*_1_ from *Bos taurus* with ubiquinone bound at the Q_i_ site (PDB code: 1NTZ).^41^ This structure was chosen for several reasons. It includes the substrate bound at the Q_i_ site, and its quality is sufficient for geometry optimization of large models using QM methods. Also, it contains resolved positions of water molecules, which, according to the literature, directly participate in the ubiquinone reduction reaction, and its Q_i_ site shows high structural similarity.^42^ When the Q_i_ sites from *B. taurus* and *R. capsulatus* are compared, subtle differences can be observed, but they do not involve the residues directly participating in the reaction. The cluster model of the Q_i_ site consisted of 334 atoms and included the following components: heme *b*_H_, ubiquinone with its hydrophobic tail truncated to one isoprene unit (as in the model of the Q_o_ site), four water molecules (in the same positions as in the 1NTZ structure), and 13 protein residues: I27^*Bt*^/L41^*Rc*^ (*Bt* and *Rc* in superscript denote *B. taurus* or *R. capsulatus*, respectively), W31^*Bt*^/W45^*Rc*^, H97^*Bt*^/H111^*Rc*^, R100^*Bt*^/R114^*Rc*^, H196^*Bt*^/H212^*Rc*^, L200^*Bt*^/F216^*Rc*^, H201^*Bt*^/H217^*Rc*^, S205^Bt^/N221^*Rc*^, N206^*Bt*^/N222^*Rc*^, F220^*Bt*^/F244^*Rc*^, Y224^*Bt*^/F248^*Rc*^, K227^*Bt*^/K251^*Rc*^, D228^*Bt*^/D252^*Rc*^. In the QM model of the Q_i_ site, the amino and carbonyl groups of the protein backbone were replaced by hydrogen atoms in the following residues: I27, W31, H97, R100, H196, L200, H201, and Y224. In F220, the Cβ atom was replaced by a hydrogen atom. The fragments S205–N206 and K227–D228 were included in the model, with their peptide bonds preserved. Accordingly, only the NH backbone moieties of S205 and K227 and the CO backbone moieties of N206 and D228 were substituted with hydrogen atoms.

Protonation states of the residues were assigned using program Propka 3.1 ^36,37^ and validated by PypKa 2.10.0.^38^ The geometry optimization of intermediates involved in the ubiquinone reduction was perfomed with the constraints imposed on atoms derived from the protein backbone, including hydrogen atoms replacing any moieties present in the whole protein structure.

### QM Methods

Due to the very large cluster models (∼400 atoms) used in this study, computational limitations rendered vibrational analysis and full transition-state characterization unfeasible^43,44^ ; therefore, constrained TS-like structures (pre-TS*) were employed to provide upper-bound estimates of the transition-state energies. All QM computations were performed using the Gaussian 16 program.^45^ The geometries of all stationary points were optimized using DFT with the B3LYP hybrid exchange–correlation functional and Grimme’s D3 dispersion correction with Becke–Johnson damping.^46,47^ Geometry optimizations were performed with the double-ζ basis set def2-SVP, while the final energies of the intermediates were computed with the triple-ζ basis set def2-TZVP, within a polarizable continuum model (PCM) characterized by a dielectric constant of 4.0 and a probe radius of 1.4 Å, to mimic the electrostatic effects of the protein environment.^48^ The dielectric constant was set to 4 to reflect the highly hydrophobic environment of the Q_o_ sites and the surroundings in cyt. *bc*_1_.^49,50^ These methods have been previously validated for computational studies of active sites involving iron–sulfur clusters and hemes.^51,52^

Since the **P3** structure relaxes to **I2** (see Figure 2) and during geometry optimization with doublet multiplicity in our model, in which heme *b*_H_ is not explicitly included, its geometry was optimized in the quartet multiplicity state, and the final energy was computed for the doublet state. The presence of the reduced heme *b*_H_ (*b*_H_^red^) is expected to decrease electron affinity of heme *b*_L_ due to electrostatic interactions and should stabilize **P3** state in respect to **I2**. To overcome limitations of the optimization algorithm associated with geometry optimization of the shallow minimum state of **P3** (in dublet multiplicity), the quartet state was used to optimize the geometry of **P3**. The results revealed that energy obtained for quartet and doublet for geometry optimized for quartet multiplicity are identical. Thus, for the spin orientations of heme *b*_L_^ox^ considered (α, β), the energy of the **P3** state, which corresponds to ferromagnetic coupling between SQ and the reduced 2Fe2S cluster, remains essentially unchanged. This is consistent with the observation that at temperature >20 K, CW EPR spectra of SQ-2Fe2S^red^ still remain unaffected despite a very fast spin–lattice relaxation rate (≫ 4 × 10^6^ s^–1^) of iron ion in heme *b*_L_.^21,53^

**Figure 2:**
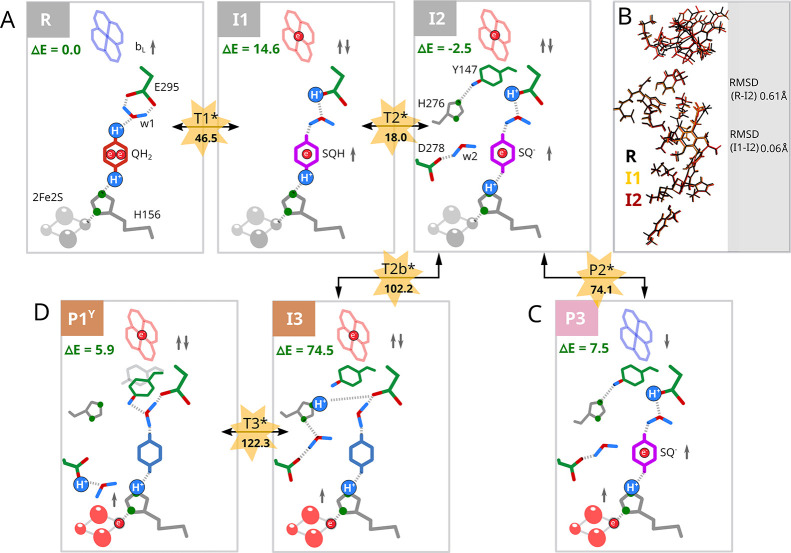
Primary steps of QBEB without involvement of ET from heme *b*_L_ to *b*_H_. (A) **R** denotes a starting state comprising heme *b*_L_^ox^ (*blue crossed-hexagon*), QH_2_ (*red hexagon*), and 2Fe2S^ox^ (*gray ovals*). The reaction progresses to **I1** and **I2** states containing heme *b*_L_^red^ (*red crossed-hexagon*) and SQH, or SQ^–^ (*magenta hexagon*), respectively. (B) Superimposed geometries of **R**, **I1**, and **I2**. (C) Evolution of **I2** to stable **P3** containing heme *b*_L_^ox^, SQ^–^, and 2Fe2S^red^ (*red ovals*), via P2*. (D) Evolution of **I2** to **P1** ^Y^ containing heme *b*_L_^red^, Q, and 2Fe2S^red^ via high energy T2b*, **I3**, and T3*. Gray arrows indicate the net spin corresponding to the redox state of the substrate and cofactors. Side chains of the protein residues are shown when their protonation and/or conformation state is significant. Important water molecules are labeled as “w1” and “w2”, and protons derived from QH_2_ in the given state are represented by blue circles. The energy barriers were estimated via the energy of pre-TS states (*yellow stars*). Energy is given in kJ/mol.

### Material Used for Experimental Sample Preparation

Equine cytochrome *c* (cyt. *c*), 2,3-dimethoxy-5-decyl-6-methyl-1,2-benzoquinone (DB), and antimycin were purchased from Sigma-Aldrich (St. Louis, MO, USA). Cyt. *bc*_1_ was isolated from *R. capsulatus* grown under semiaerobic conditions, as described previously.^54^ The isolated cyt. *bc*_1_ solution was dialyzed against reaction buffer composed of 50 mM bicine (pH 8.0), 100 mM NaCl, and 0.01% (w/w) dodecylmaltoside (DDM). DB was reduced to its hydroquinone form (DBH_2_) using sodium borohydride. Iron­(III) citrate, used as an internal standard for sample packing efficiency, was prepared immediately before the experiment by adding FeCl_3_ to a 10-fold molar excess of citric acid.

### Freeze-Quench Experiments

Nonequilibrium samples of cyt. *bc*_1_ were prepared as described previously.^55^ Briefly, freeze-quenched samples were generated using a Biologic SFM-300 stopped-flow mixer equipped with an MPS-70 programmable syringe controller and electron paramagnetic resonance (EPR) freeze–quench accessories. One syringe contained a mixture of cyt. *bc*_1_ (50 μM), antimycin (250 μM), cyt. *c* (370 μM), and Fe^3+^ citrate (10 μM). The second syringe contained a solution of DBH_2_ (390 μM) in a bicine buffer. The reaction was initiated by mixing the contents of the two syringes in a 1:1 volume ratio. The mixture was incubated in a delay line for a programmed period and then injected into an isopentane bath cooled to 170 K. The frozen droplets were collected in an EPR tube and measured immediately after preparation. Different occupancies of the Q_o_ site by quinone radical states were achieved by modulating the sample incubation time.

### Double Mixing Experiments

For the double mixing experiments shown, we used our home-built multichannel pulse high-current drivers and a custom mixer with a rotary valve, as described previously.^56,57^ Cyt. *bc*_1_ was prepared at a concentration of 80 μM in bicine buffer (as described above) and supplemented with 100 μM antimycin. Prior to mixing, portions of potassium ferricyanide were added and the oxidation level of cyt. *c*_1_ was continuously monitored spectrophotometrically. Addition of the oxidant was continued until 90% of heme *c*_1_ was oxidized. Synthetic ubiquinol (DBH_2_) and equine cyt. *c* were prepared in bicine buffer at concentrations of 180 and 300 μM, respectively. Syringes 1 and 2 contained 125 μL of cyt. *bc*_1_ solution and 125 μL of DBH_2_ solution, respectively. The solutions were mixed and incubated for 500 ms in syringe 3 to allow the enzyme to reach the state of **P3**. Then, 250 μL of the cyt. *bc*_1_ + DBH_2_ mixture was combined with 250 μL of cyt. *c* solution was aged for an additional 200 ms. Finally, the mixture containing cyt. *bc*_1_, DBH_2_, and cyt. *c* was injected into cold isopentane to stop the reaction. The pulse sequence applied in this experiment is presented in Table 1. This procedure was repeated twice using an independently isolated cyt. *bc*_1_ preparations, and each experiment was performed in triplicate.

**Table 1:** Pattern of Pulses Used for the Multichannel Pulse High-Current Drivers

start [ms]	0	500	700	705	800
duration [ms]	35	70	60	1	120
piston number	1, 3	1, 2	4		
channel				4	
power	100%	100%	70%	25%	30%

### EPR Measurements

All EPR measurements were performed using a Bruker Elexsys E580 spectrometer (Bruker, Billerica, MA, USA). X-band continuous wave EPR spectra were measured using an SHQE0511 resonator and an ESR900 cryostat (Oxford Instruments, Abingdon, UK). The helium temperature was maintained using a closed-cycle Stinger cryocooler (ColdEdge, Allentown, USA). The parameters for reduced 2Fe2S and Fe^3+^ citrate measurements were as follows: temperature of 20 K, microwave power of 2 mW, modulation amplitude of 14.36 G, conversion time of 40.96 ms, time constant of 81.92 ms, sweep time of 83.89 s, and number of scans of 1–3, depending on the signal-to-noise ratio. For the free radical signal, the following parameters differed: temperature of 200 K, microwave power of 80 mW, modulation amplitude of 6 G, and number of scans of 1–12, depending on the signal-to-noise ratio. All amplitudes of the recorded EPR spectra were normalized using the amplitude of the Fe^3+^ citrate spectra as the internal standard.

## Results and Discussion

### A: Computational Study

To understand how nature realizes QBEB at a fundamental level, we conducted QM calculations of possible enzyme-catalyzed reactions using multiple models with different redox and protonation states, based on cyt. *bc*_1_ crystallographic structures obtained from *R. capsulatus*. For clarity, we specify that in this work, QBEB is considered as a minimal cascade of internal reactions within the enzyme, leading to a stable, low-energy state containing the product Q, which corresponds to a state that can be detected experimentally as the dominant one under equilibrium conditions, i.e., upon depletion of reaction substrates. In this work, the terms “equilibrium” and “non-equilibrium” are used to refer to experimental conditions in which substrates and products are at redox equilibrium (no net catalytic turnover) and to conditions in which substrates and products have not yet reached equilibrium and the enzyme operates under continuous turnover, respectively. We consider only the primary QBEB steps, rather than the entire catalytic Q cycle. For the first time, and unlike in previous studies,^58–62^ the QM model of Q_o_ included both acceptors of electrons from QH_2_:2Fe2S and heme *b*_L_ (Figure 1D,E). In the starting state, QH_2_ forms a hydrogen bond to deprotonate H156 of the iron–sulfur protein (*R. capsulatus* numbering) and via the water molecule to E295 of cyt. *b*, at the time when immediate electron acceptors 2Fe2S and heme *b*_L_ are oxidized. Y147 of cyt. *b* adopts two conformations: away from or close to E295 (**R** in Figure 1D or **R** ^**Y**^ in Figure 1E; Figure S1). We tested the effect of protonation of H156 but in that case, no reaction was observed. The choice of deprotonated state of H156 for the enzyme–substrate complex was supported by several experimental observations suggesting that protonation of this residue at lower pH inhibits activity of cyt. *bc*_1_.^63–67^ The rationale behind selection of the starting state is explained in the SI (Section “Selection of the starting model”).

Initially, following the assumptions of the canonical QBEB, we tested the scenario in which the reaction is expected to start from **R** or **R** ^**Y**^ with protonation of H156 by C4-OH of QH_2_ (atom numbering in Figure 1F) followed by ET to 2Fe2S. Neither expected structures containing heme *b*_L_^ox^, QH^–^, protonated H156, and oxidized 2Fe2S (2Fe2S^ox^) nor those with heme *b*_L_^ox^, SQH/SQ^–^, and reduced 2Fe2S (2Fe2S^red^) could be obtained in our QM models. Instead, relocation of the proton from C4-OH to H156 is accompanied by both PT from C1-OH to E295 and simultaneous ET from QH_2_ to heme *b*_L_. This led to the intermediate containing SQ^–^, heme *b*_L_^red^, and 2Fe2S^ox^ (**I2** in Figure 2A and **I2** or **I2** ^**Y**^ in Figure S2), which appeared in a clear contradiction with predictions based on the canonical QBEB, namely, SQ formation together with 2Fe2S^red^ and heme *b*_L_^ox^. Such a state seems quite advanced in the progression of QBEB as it must have resulted from at least two PTs and one ET (protons are already on H156 and E295 and the electron is on heme *b*_L_). It thus appears that the transition from **R**/**R** ^**Y**^ to **I2**/**I2** ^**Y**^ might involve an intermediate step to be considered. Therefore, we went back to the beginning of the reaction and tested how **R** or **R** ^**Y**^ responds to the initial water-mediated PT from C1-OH to E295. Surprisingly, this PT was coupled to ET from QH_2_ to heme *b*_L_, leading to **I1** or **I1** ^**Y**^, (states containing SQH, heme *b*_L_^red^, and 2Fe2S^ox^, Figure 2A and Figure S2). The emergence of heme *b*_L_^red^ next to 2Fe2S^ox^ was an unexpected result, given that *E*_m_s of heme *b*_L_ and 2Fe2S, determined for proteins under equilibrium, suggests a higher electron affinity for 2Fe2S than heme *b*_L_. This prompted us to scrutinize the electronic structure of **R**, which was taken as the most probable state of the Q_o_-QH_2_ complex (**R** ^**Y**^ and the following **I1** ^**Y**^ were 3.8 and 17.2 kJ/mol less stable than **R** and **I1**, respectively).

#### Asymmetry in Charge and Spin Distribution in the Initial Structure

In **R**, there is a shift of electron density from QH_2_ toward heme *b*_L_, associated with a spread of unpaired α spin density (SD) over M154 and D278 of cyt. *b* toward QH_2_ (Figure 3A). At the same time, α (SFe1 = 5/2) and β (SFe2 = −5/2) SD are present on iron ions of 2Fe2S^ox^ but antiferromagnetism cancels the net spin. Shifting of electron density toward heme *b*_L_ imposes a heterogeneous distribution of the local electric potential around the quinone ring with a more negative potential at the region of C1-OH compared to C4-OH (Figure 3B, red arrow). At the same time, the electronic structure indicates that the highest occupied and lowest unoccupied molecular orbitals (HOMO and LUMO, respectively) are located on QH_2_ and heme *b*_L_, respectively (Figure 4). The charge polarization and the location of HOMO and LUMO determines direction of ET from C1-OH to heme *b*_L_^ox^ accompanied by PT to the carboxyl group of E295, resulting in state **I1** (Figure 2A and Figure S2A).

**Figure 3:**
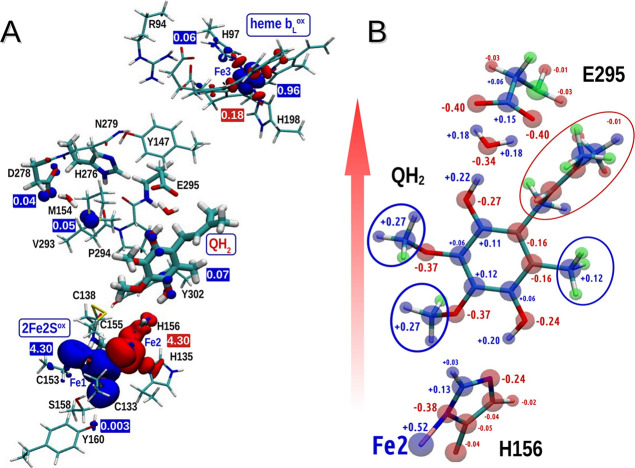
Spin and charge distribution in **R**. (A) The distribution of α (blue) and β (red) spin density in the most stable structure containing QH_2_. The numbers indicate the spin densities. (B) The charge distribution within the closest surrounding of the QH_2_ molecule. Red arrow indicates the direction of the shift of negative charge at Q_o_ toward heme *b*_L_. Red, blue, and green circles indicate atoms bearing partial negative, positive, and neutral charge, respectively. Size of the numbers reflect the magnitude of the deviation of the charge from neutral.

**Figure 4:**
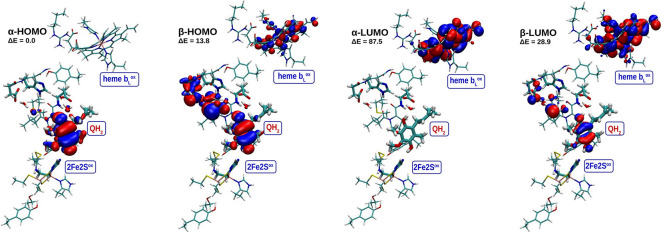
Location of HOMO and LUMO orbitals and their relative energy (with respect to the α-HOMO) in kJ/mol for the most stable structure containing QH_2_, heme *b*_L_^ox^, 2Fe2S^ox^, and deprotonated H156.

It is important to emphasize that it was not possible to obtain the **I1** state when 2Fe2S is not involved (Figures S3–S6), because in models deprived of the cluster, HOMO orbitals were no longer located on the QH_2_ molecule. On the other hand, for the models containing the 2Fe2S cluster but not heme *b*_L_, we obtained a stable state containing SQ-2Fe2S^red^ similar to previous work^58^ (see optimized geometries and HOMO, LUMO orbitals in Figure S7). This would indicate that either electron transfer from QH_2_ to 2Fe2S is exergonic, which seems incompatible with the canonical QBEB, or it is an artifact of the truncated model. If such a model truly reproduced the first QBEB step, one would expect to detect an EPR signal of SQ and/or SQ–2Fe2S^red^ under equilibrium conditions and in the cyt. *bc*_1_ mutant with the heme *b*_L_ knocked out, which is not observed.^68^ Clearly, approaches for determining initial steps of the QBEB using models containing only one cofactor at a time suffer from the *a priori* imposition of the sequence of the reactions.

Altogether, these observations indicate that reaching **I1** requires the presence of both heme *b*_L_ and 2Fe2S, despite an apparent lack of involvement of the latter in this process. Furthermore, 2Fe2S and its direct surrounding must be in the correct state, as the models of **R** variants with either protonated H156 and 2Fe2S^ox^ or deprotonated H156 without the cluster also failed to show any signs of progress to **I1** (Figure S8).

#### Reversible QBEB Stages on a Way toward Products

The state **I1** passes to **I2** by shifting H^+^ originating from the C4-OH group of SQH toward H156. The RMSD parameter comparing the positions of all atoms in **R**, **I1**, and **I2** is about 0.06 Å indicating negligible structural rearrangement upon **R ↔ I1 ↔ I2** transitions (Figure 2B).

The energy levels of **R**, **I1**, and **I2** are close to each other (0.0, 14.6, and −2.5 kJ/mol, respectively) and the estimated upper-bound barriers for **R ↔ I1** (46.5 kJ/mol) and **I1 ↔ I2** (3.4 kJ/mol) transitions are relatively small, which means that at room temperature (∼300 K corresponding to ∼3 kJ/mol) **R** and **I2** should be similarly populated. Thus, **R**, **I1**, and **I2** are considered as degenerate, coexisting states with **I1** being a transient form between **R** and **I2.**

We first searched for states containing stable product under conditions when the electron on heme *b*_L_ cannot advance to heme *b*_H_ (like in antimycin-inhibited enzyme). One tested reaction involved elongation of the H-bond between SQ^–^ and protonated H156. This resulted in unstable P2* (Figure 2C and Figure S9), which immediately relaxed back to **I2** or evolved to **P3**. The appearance of the **P3** state is notable because it contains heme *b*_L_^ox^ together with SQ and 2Fe2S^red^. Because both of these paramagnetic centers (SQ and 2Fe2S^red^) have parallel spins and are connected with H bonds, one can expect formation of an ferromagnetically coupled SQ-2Fe2S center (discussion in SI, Figure S10). The state P2* should be considered as an approximate transition state (pre-TS), with an estimated energy of +74.1 kJ/mol, between **I2** and **P3**. We note that a nearly identical geometry of nuclei in **I2** and **P3** indicates a potential contribution from the electron tunneling process, which effectively decreases the barrier between these states.

The second tested reaction involved shifting the proton away from E295 to D278. This resulted in relatively stable **P1** ^**Y**^ containing Q (Figure 2D). However, as reaching this state from **I2** is slightly endergonic and requires crossing barriers (104.7 and ∼47.8 kJ/mol, Figure 2) with a high energy state in between (**I3**, +74.5 kJ/mol, Figure S11), this process is unlikely. Consequently, when electron cannot advance from heme *b*_L_ to heme *b*_H_, the enzyme is expected to become trapped in the states **R** ↔ **I1** ↔ **I2** and **P3** without possibility to produce a low-energy product Q. This is consistent with the inhibitory effect of antimycin on the Q_o_ site.

#### Release from Degenerate States and Emergence of Products

In the further search for exergonic processes that would push QBEB toward low-energy products, we estimated the net energetic cost associated with ET from heme *b*_L_ through heme *b*_H_ to Q_i_ (Figure 5). We considered a state **I2’**, which is a result of removal of electron from heme *b*_L_^red^ in **I2**, and various states of the complementary model that mimicked the acceptance of this electron. The complementary models encompassed heme *b*_H_ and quinone at Q_i_ (details in SI and Figure S12). The results indicate that the transition from **I2** to **I2’** would be endergonic (+283.1 kJ/mol) if the complementary model was not included. However, the average energy released per electron delivered to Q_i_ (−401.1 kJ/mol) lowers energy of **I2’** to −118.1 kJ/mol (Figure 5). In this way, ET from Q_o_ to Q_i_ makes QBEB exergonic, allowing Q_o_ to depart from degenerate states **R** ↔ **I1** ↔ **I2** and **P3** (the latter state is unlikely to be formed when this ET is not blocked). The net energy released in Q_i_ drives the enzyme to overcome local barrier associated with PT from E295 to H276 (state **I3′**). The following PT from H276 to D278 is coupled to ET from SQ to 2Fe2S^ox^, which weakens the interaction between the quinone moiety and protonated H156 (the H bond elongates from 1.5 to 2.0 Å, Figures S13 and S14). This reaction is the final step of QBEB, leading to the low-energy state **P1’** ^**Y**^ (−96.3 kJ/mol in respect to **R**) containing the product (quinone).

**Figure 5:**
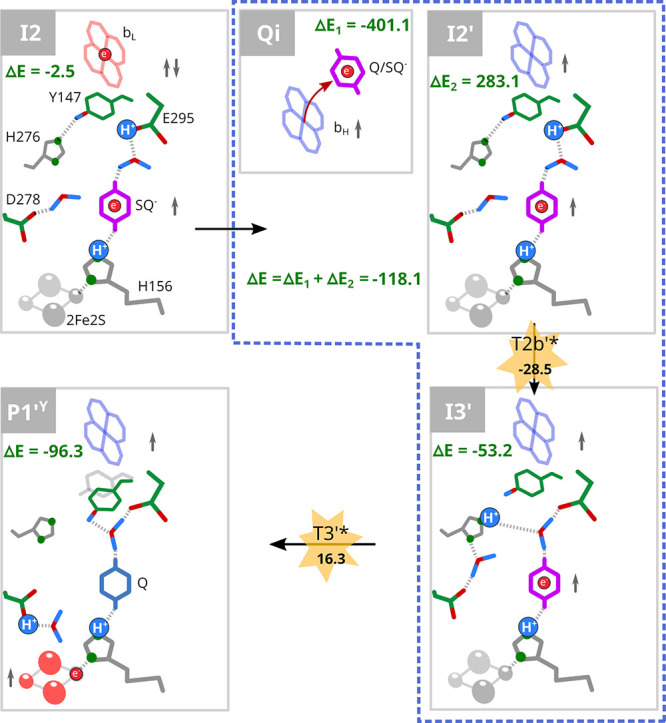
Completion of QBEB involving energy released during ET from heme *b*_H_ to Q_i_. The ET from heme *b*_L_ to heme *b*_H_ and subsequently to Q_i_ provides a highly exergonic step that resolves the **R ↔ I1 ↔ I2** degeneration (see Figure 2), driving the reaction from **I2** toward **I3′**. The energy associated with this reaction was calculated using the states **Q**_i_ and **I2’**, **I3′** considered together (*blue dashed square*). **P1’** ^**Y**^ denotes the final state of QBEB containing Q, 2Fe2S^red^, and protonated D278. The symbols are consistent with those used in Figure 2. Red arrow indicates ET. The energy barriers were estimated via energy of pre-TS states (*yellow stars*). Energy is given in units of kJ/mol.

We note that while the essence of the departure from degenerate states lies in effective removal of the proton away from the quinone ring, the exact PT path is not a determinant of this process but influences its efficiency. The process might be tolerant to mutational changes, and the residues that build the path may vary in different species.^40,69–71^ We also note that, in this mechanism, completion of QBEB does not require the motion of the ISP away from Q_o_, implicated as an inherent feature of catalysis. It is worth mentioning about recent reports on structures of cyt. *bc*_1_ for which ISP does not move during the catalytic cycle.^72^ When the motion occurs during further steps of enzymatic catalysis that follow **P1’** ^**Y**^, transferring electrons toward more remote cofactors (*c*-type hemes) further lowers the energy of the system. However, as mentioned above, these steps remain beyond consideration of this work.

Figure 6 summarizes the most probable enzyme states that constitute the pathway of QBEB inferred from energetic relations between the states obtained by QM (see Figure S15 for all the considered states). When QH_2_ bound in Q_o_ encounters 2Fe2S^ox^ and heme *b*_L_^ox^ (**R**), the negative charge from the quinol moiety initially shifts toward heme *b*_L_ (**I1**, **I2**). ET from the resulting SQ to 2Fe2S (**I3′**, **P1′** ^**Y**^) is powered by the energy released upon electron transfer from heme *b*_L_ to Q_i_ (blue line). Without this energetic input, the enzyme is trapped in states **R**, **I1**, **I2**, and **P3**, with no possibility of reaching a low-energy product state. These results highlight that productive quinol oxidation cannot be explained by a strictly sequential two-step scheme but instead requires a cooperative route initiated at heme *b*_L_^ox^.

**Figure 6:**
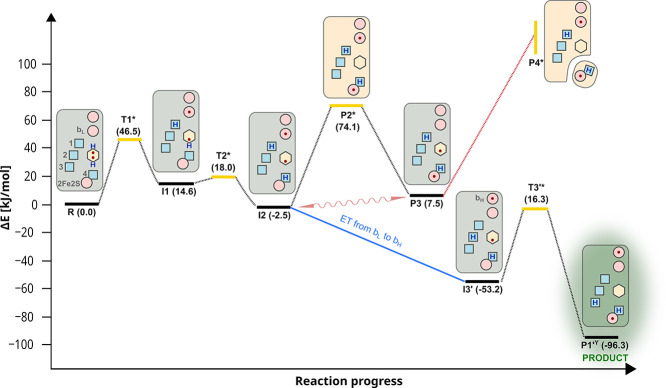
Key states of the EMET model of QBEB. Solid black lines represent relative energy levels (numbers in parentheses) of the states on the way from the substrate (**R**) to product (**P1’** ^**Y**^). Yellow solid line represents energy level of pre-TSs (T1*, T2*, T3*, and P2*). Red circles – electron acceptors; blue squares – proton acceptors 1, 2, and 3 in cyt. *b* (in *R. capsulatus* E295, H276, and D278, respectively) and 4 in ISP (histidine ligand to 2Fe2S); yellow hexagon – quinone moiety; red dots and blue “H” – electrons and protons derived from QH_2_; wavy arrow – electron tunneling; dotted lines illustrate barriers between states. Red dotted line denotes the possibility of quinone formation P4* from **P3** by elongation of the distance between the ISP and the Q_o_. The energy level of P4* increases due to uncompensated breaking interactions and thus cannot be determined precisely (yellow vertical line). Antimycin prevents oxidation of heme *b*_H_; thus, the transition from **I2** to **I3′** (blue line) and subsequently to **P1**’^**Y**^ is not possible. The green background indicates the state in which stable product–quinone is formed at the Q_o_ site.

#### Implications from QM Calculations

We performed QM calculations to explore a possible mechanism underlying the initial stages of electron bifurcation. Our motivation arose from the interpretative challenges posed by experimentally observed enzyme states, particularly under antimycin inhibition. The models employed explicitly included both immediate electron acceptors (2Fe2S^ox^ and heme *b*_L_^ox^) and proton acceptors at each reaction step. This approach avoided the predefined sequence of events inherent to the canonical QBEB models, in which the reaction is partitioned into two sequential one-electron transfers: first from QH_2_ to 2Fe2S^ox^ and then from SQ to heme *b*_L_^ox^ (see Figure S16 for general scheme summarizing the canonical QBEB). Optimized stationary points, together with EPR spectroscopic evidence, revealed an alternative to the canonical QBEB mechanism in which QBEB is initiated by electron transfer from QH_2_ to heme *b*_L_^ox^ and proceeds along an energetically favorable cooperative pathway with engagement of the integrated system of redox cofactors. To distinguish this mechanism from the canonical QBEB, we introduce the term EMET, a conceptual model that emphasizes the alternative QBEB pathway not anticipated within conventional the canonical QBEB assumptions.

An important aspect of the EMET mechanism is that the initial shift of negative charge toward heme *b*_L_^ox^ does not occur in models of **R** deprived of 2Fe2S (Figures S3–S6). In this case, although the LUMO orbitals are still present on heme *b*_L_^ox^, the HOMO orbitals do not localize on the QH_2_ molecule. This result automatically precludes the ET process in such a model. Additionally, this also eliminates the possibility of reproducing the sequence of reactions identified in our *ab initio* modeling by splitting it into two separate one-electron reactions, since in that case, we would, *de facto*, be calculating an irrelevant system. Nevertheless, we checked whether the sequence of reactions predicted by models built on assumptions of the canonical QBEB could be reproduced by an analogous splitting into two reactions. In this case, we tested the possibility of the first ET from QH_2_ to 2Fe2S^ox^ in a model of **R** deprived of heme *b*_L_. We found that SQ along with 2Fe2S^red^ were formed but, contrary to the assumptions of the canonical QBEB, this state has lower energy than **R**. This meant that the process turned out to be exergonic, leading to the formation of a stable SQ at Q_o_. In contrast, the complementary reaction of the second ET from SQ to heme *b*_L_^ox^ (reproduced in **P3** deprived of 2Fe2S) was found to be endergonic, introducing a high energy state that cannot be easily reached. As the electron does not reach heme *b*_L_, further ET toward Q_i_ is not possible, and the system is expected to become trapped in a state with relatively stable SQ, potentially detectable by EPR under equilibrium conditions. This outcome is inconsistent with both the canonical QBEB assumptions and experimental observations. Thus, splitting QBEB into two separate ET reactions appears to introduce artifacts, either preventing the initial reaction or producing a stable SQ. Therefore, the occurrence of QBEB must be viewed as an inherent emergent property of QH_2_ oxidation at Q_o_.

### B. Experimental Validation and Predictions Inferred from the EMET Model

Our results revealed a fundamentally new picture of the primary steps of QBEB catalyzed by cyt. *bc*_1_, substantially different from the canonical QBEB. While it is virtually not possible to point to a single experimental observation that would directly validate this mechanism, we highlight several key predictions of the EMET model along with corresponding experimental observations that, taken together, support the proposed mechanism.

#### Prediction 1

According to the EMET model, antimycin-inhibited cyt. *bc*_1_ exposed to substrates becomes trapped in the states **R** ↔ **I1** ↔ **I2** ↔ **P3** (Figure 6). Among these states, **P3**, containing SQ^–^ and 2Fe2S^red^ with parallel spins, appears to be the most characteristic spectroscopically detectable species. The structural proximity of these two centers, connected by an H bond, provides a strong argument for the origin of the spin-coupled SQ-2Fe2S^red^ EPR signal.^21,22,73^ This signal exhibits a rhombic spectrum arising from spin–spin exchange interaction with an estimated frequency of ∼3.6 GHz, producing the most prominent transition at *g* = 1.94 (Figure 7A, green), clearly distinguishable from the 2Fe2S^red^ signal at *g* = 1.90 (Figure 7A, black). It can be further suggested that the amplitude of the SQ-2Fe2S^red^ signal should correlate with the amplitude of the SQ radical signal, posed by **I1** and/or **I2** if the system is in the stationary state during the turnover. Therefore, one would expect the SQ signal to be detectable by EPR in the same samples containing SQ-2Fe2S^red^. Indeed, several reports describe distinct SQ signals under nonequilibrium conditions in antimycin-inhibited cyt. *bc*_1_ including ours.^21–25,74,75^ In this work, we also observed an SQ signal at temperatures above 150 K, together with SQ-2Fe2S^red^ at 20 K (Figure 7B). Interestingly, these two signals were found to be linearly correlated, in agreement with expectations derived form the EMET model. While the SQ–2Fe2S^red^ signal provides a specific signature of spin coupling between two defined cofactors, assigning the SQ signal itself to **I1** and/or **I2** remains inherently challenging. At this stage, we cannot rule out the possibility that the detected SQ arises from a thermally activated process related to the SQ–2Fe2S^red^ state.

**Figure 7:**
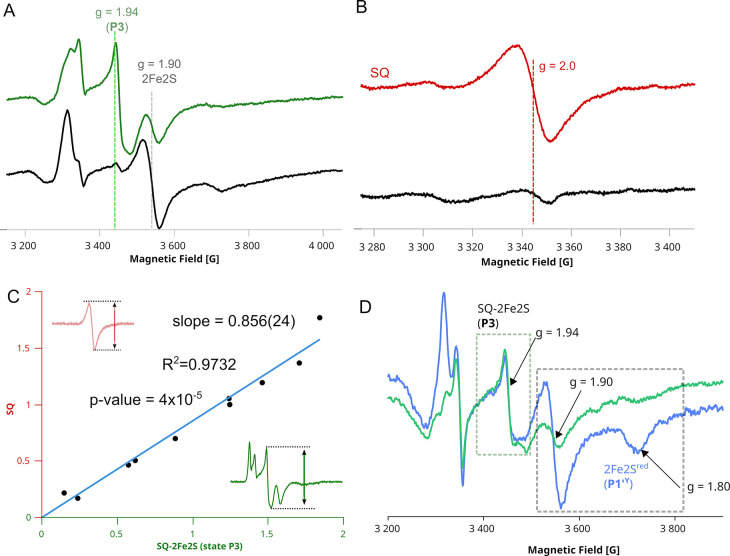
Experimental data obtained for antimycin-inhibited cyt. *bc*_1_. (A) EPR spectra of 2Fe2S^red^ and coupled SQ–2Fe2S^red^ (**P3**) measured at 20 K for samples prepared under equilibrium (black) and nonequilibrium conditions (green; 1 s after substrate addition). Dashed vertical lines indicate the main EPR transitions 2Fe2S^red^ (gray) and SQ–2Fe2S^red^ (green). (B) SQ radical signal measured at 200 K in the same samples as in panel (A), under equilibrium (black) and nonequilibrium (red) conditions. (C) Correlation between the amplitudes of SQ and *g* = 1.94 EPR signals for samples prepared at different time points after mixing with substrates. (D) Double-mixing EPR experiment showing spectra of enzyme preset to **P3** followed by mixing with buffer (*blue*) or with an additional portion of oxidized cyt. *c* (*green*). Gray and green dashed boxes highlight spectral ranges corresponding to 2Fe2S^red^ with bound Q and SQ–2Fe2S^red^ (**P3**), respectively. Arrows indicate the *g* = 1.94 transition of SQ-2Fe2S^red^ and 1.90 and 1.80 for g_*y*_ and g_*x*_ of 2Fe2S^red^, respectively.

#### Prediction 2

When the reaction reaches equilibrium after substrate exhaustion, both the SQ and SQ–2Fe2S^red^ EPR signals disappear (Figure 5A,B; red and black, respectively). This provides clear evidence that they originate from species formed exclusively under nonequilibrium conditions and therefore represent short-lived, experimentally detectable states that are populated only under continuous turnover conditions. Moreover, EMET predicts that **P3** occurs in enzyme molecules in which heme *b*_L_ remains oxidized. Accordingly, the signal at *g* = 1.94 should be accompanied by the heme *b*_L_^ox^ signal, a result consistently reproduced in experiments.^21,22^

#### Prediction 3

The transitions between **I2** and **P3** are relatively slow as they require crossing **P2** *, which lies ∼76.6 kJ/mol above **I2** and 66.6 kJ/mol above **P3**_._ This barrier corresponds to the rate constant of approximately 1/s.^76^ The experimentally obtained rate of growth of the SQ-2Fe2S^red^ signal (Figure 8 green) is ∼4.7/s, which, according to the Eyring equation, corresponds to an energy barrier of ∼67.0 kJ/mol. If the observed growth rate of the SQ–2Fe2S^red^ signal indeed reflects the internal barrier-crossing step rather than substrate diffusion, then the close match with the calculated values, within this assumption, supports the reliability of the energy profile in Figure 6.

**Figure 8:**
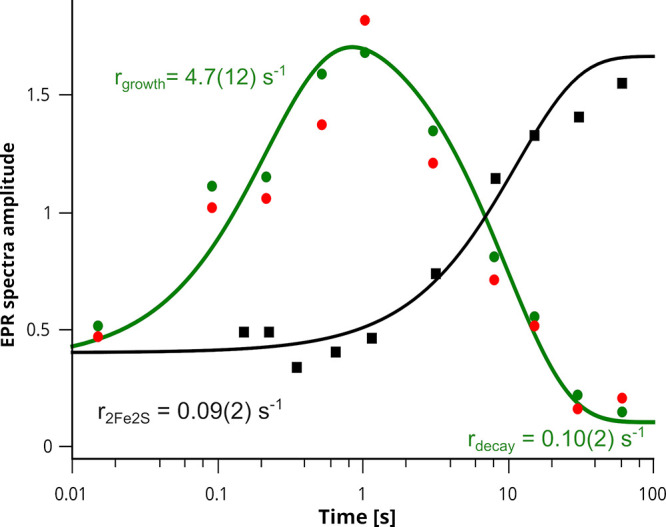
Time dependence of EPR signals in antimycin-inhibited cyt. *bc*_1_. Green and red circles represent the amplitudes of SQ–2Fe2S^red^ (*g* = 1.94) and SQ (*g* = 2.0), respectively. Black squares denote the amplitude of 2Fe2S^red^ with a Q bound at the Qo site. The solid green line corresponds to a biexponential fit (growth and decay) of the SQ–2Fe2S^red^ signal, while the solid black line represents a single-exponential fit of the 2Fe2S^red^ signal.

We further propose that a tunneling process (ET exchange between 2Fe2S, Q, and heme *b*_L_) may contribute to the **I2** ↔ **P3** transitions, effectively lowering this barrier.

The observed decrease of the SQ-2Fe2S^red^ signal at the rate of ∼0.1/s is explained by a spontaneous heme *b*_H_^red^ oxidation. It reopens the possibility of ET from heme *b*_L_ to *b*_H_ and progression toward the final **P1’** ^**Y**^. As a consequence, one may observe a decrease in amplitude of 2Fe2S^red^ with Q formation at Q_o_ at the same rate as SQ-2Fe2S^red^ decay (Figure 8 black). This rate is comparable to antimycin-inhibited turnover of cyt. *bc*_1_.

#### Prediction 4

The relatively slow predicted kinetics of **P3** formation, together with the parallel spins in SQ and 2Fe2S^red^, provide strong arguments against the possibility that SQ–2Fe2S^red^ arises as the initial step of QBEB resulting from electron transfer from QH_2_ to 2Fe2S^ox^. In particular, further transition of **P3** *via* the high-energy P4* intermediate would yield a product at Q_o_ and complete QBEB. According to the canonical QBEB, such a process would entail the risk of short-circuit reactions during subsequent QH_2_ oxidation to SQ because heme *b*_L_ (remaining reduced from the previous cycle) could rapidly rereduce SQ back to QH_2_. To avoid such short circuits, the enzyme must minimize this pathway, for instance, by making the **P3** → P4* transition highly unfavorable. In Figure 7D, we show the effect of blocking 2Fe2S^red^ oxidation in **P3**. When antimycin-inhibited cyt. *bc*_1_ was mixed with a substoichiometric amount of substrates, the system reached a stationary state (that can be ascribed to **P3**) after 1 s. At this stage, the oxidizing capacity (oxidized cyt. *c*) was nearly exhausted. Under these conditions, a fraction of the enzyme contained SQ–2Fe2S^red^ (Figure 7D, blue spectrum, *g* = 1.94), while another fraction contained reduced 2Fe2S^red^ with Q bound at Q_o_ (Figure 7D, blue spectrum, *g* = 1.90). The Q molecule bound at the Q_o_ site is evidenced by the *g* = 1.80 characteristic transition.^77^ Upon mixing the sample with an extra portion of cyt. *c* (increasing oxidizing power), the 2Fe2S^red^ signal decreased dramatically upon oxidation (Figure 7D, green spectrum), whereas the signal corresponding to **P3** remained unchanged. This provides strong suggestion that in the **P3** state, ISP head domain cannot move out from the Q_o_ site and thus 2Fe2S^red^ cannot undergo oxidation by ET to heme *c*_1_ and further to cyt. *c*. This means that a stable product Q cannot be generated at the Q_o_ and thus the transition from **P3** to P4* is not possible.

#### Prediction 5

The existence of **P3** resembles a “dead-end” branch in the QH_2_ oxidation pathway, blocking Q_o_ and reducing enzymatic activity from QH_2_ oxidation pathway, blocks Q_o_ and reduces the enzymatic activity from ∼400/s to approximately 4/s.^78^ This blocking effect may explain why antimycin inhibits cyt. *c* reduction by cyt. *bc*_1_. In the absence of the inhibitor, when heme *b*_H_ can accept an electron from heme *b*_L_, **I1** and **I2** remain transient, whereas **P3**, being kinetically isolated, does not accumulate to significant levels. Consequently, the paramagnetic signatures of **I1**, **I2**, and **P3** should remain undetectable by EPR under equilibrium conditions or in the noninhibited enzyme. This expectation has been confirmed by previous experiments.^79^

#### Prediction 6

Formation of **P3** requires prior ET to heme *b*_L_ (i.e., transition from **R** to **I1**/**I2**). Therefore, a mutation that disrupts the normal redox activity of heme *b*_L_ should eliminate **P3**. Indeed, the spectroscopic signature of **P3** was not detected in a mutant with impaired heme *b*_L_ function, caused by substitution of one of its axial histidine ligands with asparagine.^68^

#### Prediction 7

It can be proposed that the energy released during ET from heme *b*_L_ to Q_i_ facilitates the movement of the ISP, thereby enhancing the probability of rapid ET from 2Fe2S^red^ to cyt. *c*_1_. Indeed, although indirect, experimental evidence supporting the existence of such a mechanism has been reported.^80^

#### Large Models *vs* Transition States Estimation

Several attempts have been made to calculate TS states in modeling the QBEB; however, these models were limited only to one of the active redox cofactors. Thus, they usually included the QH_2_ and 2Fe2S^58–62^ or SQ and heme *b*_L_^ox^ at a time imposing the reaction following assumption of the canonical QBEB.^58^ We employed large cluster models (∼400 atoms) because dividing the system into smaller fragments centered on individual cofactors led to qualitatively different electronic states, resulting in a biased energetic landscape (SI, Figures S3–S8). However, the use of such large QM models renders full transition-state characterization impractical due to the prohibitive cost and numerical challenges associated with evaluating the full Hessian matrix (∼1200 × 1200 in the present case).^43^ Consequently, constrained TS-like structures (pre-TS) were used to obtain conservative upper-bound estimates of the activation barriers. In standard transition-state search protocols, pre-TS geometries serve as initial guesses for TS optimization, which rely on vibrational analysis to locate a first-order saddle point on the potential energy surface; full optimization typically yields a relaxed TS structure at a lower energy than the initial pre-TS geometry.

#### Equilibrium Redox Potentials *vs* State-Dependent Electron Affinities of Cofactors Engaged in QBEB

According to EMET, the catalytic reaction is initiated by concerted PT from QH_2_ to E295 and ET from QH_2_ to heme *b*_L_. Therefore, the kinetics of this process should be described within the framework of proton-coupled electron transfer (PCET) theory for which the conceptual framework was developed by Hammes-Schiffer and co-workers.^81,82^ In the current work, due to the size of the cluster models and computational limitations, we focused on the energetic relationships between the stationary points on the potential energy surface that define the EMET mechanism.

This reaction mentioned above has never been considered by the canonical QBEB, as in the framework of equilibrium electrochemical theory, the *E*_m_ of heme *b*_L_ appears too low for an effective withdrawal of electron from QH_2_. Reconciliation of this apparent contradiction lies in a postulation that values of redox potentials of cofactors determined under equilibrium might not be relevant when describing individual each step of the reaction but rather reflect the final thermodynamic relationships between the reduced and oxidized populations of all redox elements once the system has reached equilibrium and the reaction is complete. While this statement may seem controversial, it does not challenge the general validity of Marcus theory.^83^ Rather, it allows us to consider that under certain transient conditions present in cyt. *bc*_1_ (and possibly in other multicofactor enzymes), additional dynamic factors, such as charge separations due to ET and PT, may effectively modulate electron affinity around particular redox cofactors fixed within a protein environment of a relatively low dielectric constant. This modulation may arise from dynamic changes in the protonation states of amino acid residues and in the redox states of interacting cofactors during the course of the reaction. Indeed, previous studies have shown that reduction of one heme can lower the redox potential of an adjacent heme through Coulombic interactions by tens or even hundreds of millivolts.^84^ Such changes in electrostatic interactions often lead to splitting or stretching of Nernst curves (due to a lowering of the *n* parameter below 1) measured for cofactors spatially constrained by the protein structure.^85,86^ The effect of such interactions has also been demonstrated for electron distribution in the dimeric cyt. *b*_6_ *f*.^87^ In the case of hemes *b* at distances similar to those in cyt. *bc*_1_, shifts of up to ±100 mV should be expected.^30,84^ Moreover, protonation of H156 near 2Fe2S can shift the cluster redox potential from approximately −120 to +300 mV.^88^ The latter two examples have direct relevance to the description of transient conditions of QBEB.

Considering that the determined *E*_m_ for heme *b*_L_ corresponds to conditions where heme *b*_H_ is already reduced and for 2Fe2S^red^ where protonation of H156 has already occurred, these values clearly do not reflect the state of the active enzyme–substrate complex in which QBEB begins. At the onset of QBEB, the electron affinities of heme *b*_L_^ox^ and 2Fe2S^ox^ may be relatively similar from the ‘QH_2_ point of view’, because at this stage, heme *b*_H_ is oxidized and H156 at the 2Fe2S cluster is deprotonated (protonation prevents the reaction). This effect likely elevates the electron affinity of heme *b*_L_, lowering the energy levels of **I1** and **I2**. Consequently, a large potential separation between Q/SQ and SQ/QH_2_ is not required at the early stages of QBEB. After bifurcation is completed and equilibrium is established, the system adopts a state in which heme *b*_L_ and 2Fe2S exhibit potentials of approximately −120 mV and +300 mV, respectively, as typically determined from equilibrium redox titrations.^89^ The canonical QBEB, using these values to describe the initial stages, would require introducing a large split in the redox potentials of the Q/SQ/QH_2_ triad and, associated with this assumption, an alternating sequence of uphill and downhill steps. Consequently, the canonical QBEB faces a mechanistic issue of having to put additional constraints that would explain prevention of potential quinone-mediated short circuiting of the system. EMET, proposing a relatively flat energy profile for QBEB without alternating uphill/downhill steps, is devoid of this concern.

The EMET model presented here considers the QBEB mechanism catalyzed by cyt. *bc*_1_ at a fundamental level, providing a comprehensive explanatory framework for both current and future experiments. We believe that applying this model and moving beyond the canonical QBEB concept allow several previously difficult-to-interpret observations to be clarified, and those that appeared contradictory can now be unified. As proposed, the concept of transient changes in electron affinities may apply not only to cyt. *bc*_1_ but also to other multicofactor enzymes; future experiments should aim to confirm or falsify this model.

## Supplementary Material



## Data Availability

The experimental data supporting the findings of this study have been deposited in the RODBUK Cracow Open Research Data Repository under https://doi.org/10.57903/UJ/LVJICQ.

## Notes

Selection of the starting model; the relation between **I2**, **P2***, and **P3**; and a complementary model mimicking ET from heme *b*_L_ to the Q_i_ site; three optimized structures of the Q_o_ site containing QH_2_, considered as potential starting points for QBEB (Figure S1); the most stable optimized structures containing SQH and SQ^–^ at the Q_o_ site (Figure S2); optimized structures containing substrate and heme *b*_L_^ox^ but lacking the 2Fe2S housing fragment, including spin densities and HOMO and LUMO orbitals (Figures S3 and S4); optimized structures containing substrate, heme *b*_L_^ox^, and the H156 ligand to 2Fe2S but without the cluster, including spin densities and HOMO and LUMO orbitals (Figures S5 and S6); structure **R** optimized without heme *b*_L_, its ligands and residue R94 (Figure S7); structures containing QH_2_ (**R** ^**H**^) and QH^–^ (**I1** ^**H**^) complexes optimized with the model containing protonated H156 (Figure S8); optimized structures evolving from **I2** under conditions of blocked ET from heme *b*_L_ to *b*_H_ (Figure S9); a schematic representation of the electronic configurations of **R**, **I2**, and **P2*** involved in the formation of **P3** (Figure S10); the most stable structures considered after formation of **I2** under conditions of blocked ET from heme *b*_L_ to b_H_ (Figure S11); optimized structures of the model encompassing heme *b*_H_ and the Q_i_ site (Figure S12); the most stable structures with Y147 rotated toward H276 obtained by removing the electron from heme *b*_L_ (Figure S13); counterpart structures to those shown in Figure S13 but with Y147 rotated toward the ring of Q or SQ^–^ (Figure S14); energy levels of the optimized states considered in the DFT calculations of the QBEB process (Figure S15); and general scheme illustrating the canonical QBEB (Figure S16) (PDF)
